# Editorial: Characterization of biotherapeutic products

**DOI:** 10.3389/fbioe.2022.993262

**Published:** 2022-09-27

**Authors:** Anurag S. Rathore, C. Mark Smales, Andras Guttman

**Affiliations:** ^1^ Indian Institute of Technology Delhi, New Delhi, India; ^2^ School of Biosciences, Division of Natural Sciences, University of Kent, Canterbury, United Kingdom; ^3^ Horváth Csaba Memorial Laboratory of Bioseparation Sciences, Research Center for Molecular Medicine, Faculty of Medicine, Doctoral School of Molecular Medicine, University of Debrecen, Debrecen, Hungary

**Keywords:** biotherapeutics, analytical characterisation, biosimilarity assessment, critical quality attributes (CQAs), post translational modifications (PTMs), virus like particles (VLPs)

Characterization of biotherapeutic products is a key aspect and requirement of their successful development and commercialization. The complexity of biotech products and processes, alongside our limited understanding of how quality attributes that define a biotherapeutic impact the product’s safety and/or efficacy in the clinic remain major impediments to more predictive bioprocess development that manufacturers and regulators face. Recombinant biotherapeutic proteins are large, complex molecules and require sophisticated analytical and functional characterization methods to define their critical quality attributes and to understand how these are influenced by process and cell host changes.

In view of the above, this Research Topic aims to describe current best analytical characterisation practices of biotherapeutic products and how these can be utilised to enhance yield, quality and safety of such products. Nupur et al. offer a review of key advancements in the global regulatory landscape with respect to biosimilar approvals and also catalogue biosimilarity assessment studies for recombinant DNA products available in the public domain (Nupur et al.). Recent advancements in analytical methods, orthogonal techniques, and platforms for biosimilar characterization are covered. The review offers a comprehensive catalogue for published biosimilarity assessment studies with details on the analytical platform(s) used and critical quality attributes (CQAs) covered for multiple biotherapeutic products.

Characterization of biotherapeutic products is known to offer challenges, originating from the fact that these are extremely complex products, in particular mAbs. Chouquet et al. present recombinant production of two IgM models (IgM617 and IgM012) in pentameric as well as hexameric states and the evaluation of their polymer distribution using different biophysical methods (analytical ultracentrifugation, size exclusion chromatography coupled to multi-angle laser light scattering, mass photometry, and transmission electron microscopy) (Chouquet et al.). Each IgM construct is defined by a specific expression and purification pattern with different sample quality. Nevertheless, both purified IgMs were able to activate complement in a C1q-dependent manner. Importantly, BioLayer Interferometry (BLI) was used for characterizing the kinetics of C1q binding to recombinant IgMs. The authors show that recombinant IgMs possess similar C1q-binding properties as IgMs purified from human plasma. Further, Singh and Lee employed a two-step strategy for targeted glycan analysis of a mAb expressed in Chinese hamster ovary (CHO) cells (Singh and Lee). The first step was to create a custom library of the glycans of interest independent of glucose units values (thereby eliminating the need for a calibration curve) and instead uses accurate mass and retention time (RT) as primary search variables. The second step is to perform targeted glycan screening using the custom-built library. The developed workflow has been applied to the targeted glycan analysis of a mAb expressed in CHO for 1) cell line selection, 2) characterizing the day-wise glycan evolution in a model mAb during a fed-batch culture, 3) assessing the impact of different media conditions on glycosylation, and 4) evaluating the impact of two different process conditions on glycosylation changes in a model mAb grown in a bioreactor. Taken together, the data presented in this study provides insights into the sources of glycan heterogeneity in a model mAb that are seen during its commercial manufacturing.

Coghlan et al. address the important topic of post translational modifications (PTMs), in particular shuffled disulfide bonds (Coghlan et al.). This quality attribute has been linked to decreased potency and increased immunogenicity of protein therapeutics. To gain deeper understanding the authors designed and further optimized a semi-automated LC-MS/MS method for disulfide bond characterization on two IgG1 protein therapeutics—rituximab and bevacizumab. They also compared originator vs. biosimilar versions of the two therapeutics to determine if there were notable variations in disulfide shuffling and overall degradation between the originator and biosimilar drug products. Their data exhibited differences in how the two proteins degraded with bevacizumab demonstrating an increase in degradation whilst rituximab maintained the same level throughout the incubation. Across all methods, the originator and biosimilar drugs performed similarly.

Novel analytical methods and approaches are central to analytical characterization of biotherapeutic products. Ling et al. present a method based on Raman spectroscopy that combines extreme point sort transformation with a long short-term memory (LSTM) network algorithm for identification of therapeutic mAbs (Ling et al.). A total of 15 therapeutic mAbs were used in this study. The results indicated that the present method had good robustness against spectral peak drift, random noise and fluorescence interference. The authors conclude that the extreme point sort transformation combined with the LSTM network algorithm enables the characteristic extraction of the therapeutic mAb Raman spectrum and that the proposed method is suitable for rapid identification of therapeutic mAbs. Further, Szabo et al. introduce a capillary gel electrophoresis based workflow for 1) size heterogeneity analysis to determine the presence/absence of the non-glycosylated heavy chain (NGHC) fragment (SDS-CGE), 2) capillary gel isoelectric focusing for possible N-glycosylation mediated charge heterogeneity determination, e.g., for excess sialylation and finally, 3) capillary gel electrophoresis for N-glycosylation profiling and sequencing (Szabo et al.). Their results show the presence of negligible amount of non-glycosylated heavy chain (NGHC), while 25% acidic charge variants were detected. Comprehensive N-glycosylation characterization revealed the occurrence of approximately 8.2% core-afucosylated complex and 17% galactosylated N-linked oligosaccharides, suggesting the possible existence of antibody dependent cell mediated cytotoxicity (ADCC) effector function in addition to the generally considered neutralizing effect of bamlanivimab, the first anti-spike neutralizing monoclonal antibody, which got an emergency use authorization from the FDA for COVID-19 treatment.

Viruses and virus like particles (VLPs) seem to present challenges during their analytical characterization. Carvalho et al. evaluated several biophysical and biochemical methods for thorough characterization of monovalent and pentavalent influenza virus like particles (VLPs) from diverse groups (A and B) and subtypes (H1 and H3) produced in insect cells using the baculovirus expression vector system (IC-BEVS) (Carvalho et al.). Particle size distribution and purity profiles were monitored during the purification process using two complementary technologies—nanoparticle tracking analysis (NTA) and tunable resistive pulse sensing (TRPS). VLP surface charge at the selected process pH was also assessed by this last technique. The morphology of the VLP (size, shape, and presence of hemagglutinin spikes) was evaluated using transmission electron microscopy. Circular dichroism was used to assess VLPs’ thermal stability. Total protein, DNA, and baculovirus content were also assessed. This study shows robustness and generic applicability of the tools and methods evaluated, independent of VLP valency and group/subtype. Further, Powers et al. present a detailed characterization study that was conducted to enable understanding of a pivotal physicochemical test for adenoviral based therapeutics (Powers et al.). Anion-exchange high performance liquid chromatography (AEX-HPLC) was used to measure viral particle concentration, product purity and surface charge in a high-throughput manner. During product development of an adenoviral-based therapeutic, an accelerated stability study was performed and showed changes in each of the AEX-HPLC reportable attributes. These changes also correlated with a decrease in product infectivity prompting a detailed characterization of the impurity and mechanism of the surface charge change. Characterization experiments identified the impurity as a free hexon trimer, suggesting that capsid degradation could be contributing to both the impurity and reduced particle concentration. Additional mass spectrometry characterization identified deamidation of specific hexon residues to be associated with the external surface charge modification observed upon thermal stress conditions. To demonstrate a causal relationship between deamidation and surface charge changes observed by AEX-HPLC, site-directed mutagenesis experiments were performed. Through this approach, it was concluded that deamidation of asparagine 414 was responsible for the surface charge alteration observed in the AEX-HPLC profile but was not associated with the reduction in infectivity. Finally, Mullins et al. present results from characterization of recombinant chimpanzee adenovirus C68 low and high-density particles (Mullins et al.). The authors observed differential infectivity and product yield between two recombinant chimpanzee adenovirus C68 constructs whose primary difference was genome length. To determine a possible reason for this outcome, they characterized the proportion and composition of the empty and packaged capsids. Both analytical ultracentrifugation (AUC) and differential centrifugation sedimentation (DCS, a rapid and quantitative method for measuring adenoviral packaging variants) were employed for an initial assessment of genome packaging and showed multiple species whose abundance deviated between the virus builds but not manufacturing campaigns. Identity of the packaging variants was confirmed by charge detection mass spectrometry (CDMS), the first known application of this technique to analyze adenoviral species. The empty and packaged capsid populations were separated via preparative ultracentrifugation and then combined into a series of mixtures. These mixtures showed that the oft-utilized denaturing A260 adenoviral particle titer method will underestimate the actual particle titer by as much as three-fold depending on the empty/full ratio. In contrast, liquid chromatography with fluorescence detection proved to be a superior viral particle titer measurement methodology.

Overall, we expect this Research Topic to be of interest to those involved in characterization of biotherapeutic products.

